# Neurodegenerative Disease: What Potential Therapeutic Role of Acid-Sensing Ion Channels?

**DOI:** 10.3389/fncel.2021.730641

**Published:** 2021-10-08

**Authors:** Dalila Mango, Robert Nisticò

**Affiliations:** ^1^Laboratory of Pharmacology of Synaptic Plasticity, European Brain Research Institute, Rome, Italy; ^2^School of Pharmacy, University of Rome “Tor Vergata”, Rome, Italy

**Keywords:** ASIC1a, neurodegenerative disease, neuroinflammation, pH shift, acidosis

## Abstract

Acidic pH shift occurs in many physiological neuronal activities such as synaptic transmission and synaptic plasticity but also represents a characteristic feature of many pathological conditions including inflammation and ischemia. Neuroinflammation is a complex process that occurs in various neurodegenerative diseases such as Alzheimer’s disease, Parkinson’s disease, multiple sclerosis, and Huntington’s disease. Acid-sensing ion channels (ASICs) represent a widely expressed pH sensor in the brain that play a key role in neuroinflammation. On this basis, acid-sensing ion channel blockers are able to exert neuroprotective effects in different neurodegenerative diseases. In this review, we discuss the multifaceted roles of ASICs in brain physiology and pathology and highlight ASIC1a as a potential pharmacological target in neurodegenerative diseases.

## Introduction

The maintenance of cytosolic pH in its physiological range is required for normal neuronal activity, and even minor alterations can have serious consequences. Under normal physiological conditions, intra- and extracellular pH is maintained between 7.0 and 7.3; however, normal neuronal activity causes local pH changes (Chen and Chesler, 1992; Chesler and Kaila, 1992; Makani and Chesler, 2007; Magnotta et al., 2012). In the mammalian central nervous system (CNS), increased neuronal activity and the intense synchronous synaptic activity occurring in the synaptic plasticity process alter pH, producing alkalinization followed by acidification (DeVries, 2001; Du et al., 2014).

Under pathological conditions, including ischemic strokes, seizures, and inflammation associated with several neurodegenerative diseases, the pH shifts toward a persistent acidification that is correlated with neuronal hyperexcitability (Chiacchiaretta et al., 2017).

Neuroinflammation within the brain or spinal cord is mediated by the release of cytokines, chemokines, reactive oxygen species produced from resident microglia and astrocytes, endothelial cells, and peripherally derived immune cells (DiSabato et al., 2016). In the last decades, correlation between neuroinflammation and neurodegeneration has become increasingly solid in the light of accumulating evidence gathered on Alzheimer’s disease (AD), Parkinson’s disease (PD), and multiple sclerosis (MS) (Salter and Stevens, 2017).

Several studies in animal models and in humans have demonstrated that neurodegenerative diseases, characterized by a persistent inflammatory state, are associated with a significant reduction in the brain pH (Amor et al., 2010; Tyrtyshnaia et al., 2016; Decker et al., 2021), suggesting that neuroinflammation *per se* affects brain pH level. All this evidence has prompted the investigation of the role of acid-sensing ion channels (ASICs) in neurodegenerative diseases. The present work provides an overview of the possible roles of ASIC1a in neurodegenerative conditions and highlights this protein as a new potential therapeutic target.

## Acid-Sensing Ion Channel Channels: pH Sentinels in the Brain

**FIGURE 1:**
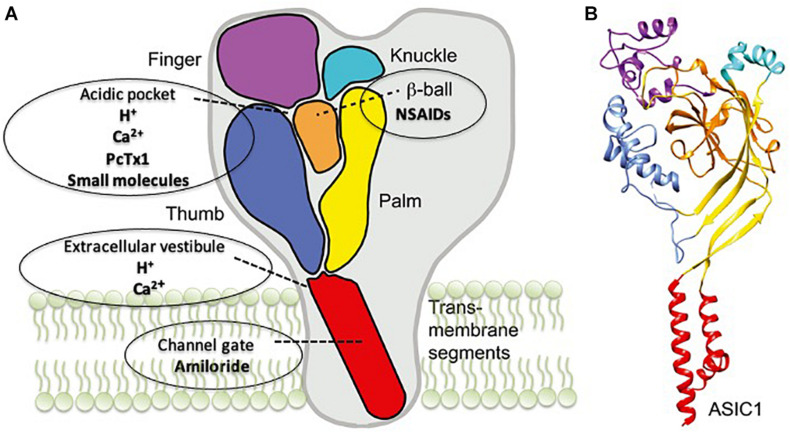
Acid-sensing ion channel 1a (ASIC1a) structure and the binding sites of its modulators. **(A)** A schematic view of the ASIC1 subunit highlighting the different domains: finger (purple), knuckle (turquoise), β-ball (orange), palm (yellow), and thumb (blue) and transmembrane domains (red). The binding sites for the different drugs are also indicated. **(B)** Crystal structure of an ASIC1 subunit obtained from chicken ASIC1 binding Mit-Tx (Baconguis et al., 2014). The domains are colored as in panel **A**. Readapted from Boscardin et al. (2016).

ASICs belong to a class of voltage-insensitive cation channels, which are widely expressed in the central and peripheral nervous systems (Sherwood et al., 2012; Vullo and Kellenberger, 2020; Rook et al., 2021). Today, it is well accepted that protons (H^+^) can act as neurotransmitters through the specific activation of proton-sensing channels, and these channels were cloned and named for the first time as ASICs in 1997 (Waldmann et al., 1997). Thereafter, many studies have investigated their structure, expression, and localization, and many experimental ligands, to explore and understand the role and function of ASIC in physiological and pathological processes, were developed (Figure 1; Chesler, 2003; Mango and Nisticò, 2020; Rook et al., 2021). Among six different mammalian protein subunits that have been cloned (ASIC1a, ASIC1b, ASIC2a, ASIC2b, ASIC3, and ASIC4), ASIC1 and ASIC2 are predominant subunits in the CNS (Waldmann et al., 1997; Chen et al., 1998; Bässler et al., 2001). Each ASIC subunit has two transmembrane domains and a large extracellular domain enriched with acidic residues. ASICs can be assembled as either homotrimers or heterotrimers to form functional channels (Bassilana et al., 1997; Babinski et al., 1999; Jasti et al., 2007; Gonzales et al., 2009; Magnotta et al., 2012; Rook et al., 2021).

The most important characteristic of ASICs is the high pH sensitivity (Wemmie et al., 2013) making them the best candidates for pH sentinels in the brain that can detect and respond to small pH variations (Papalampropoulou-Tsiridou et al., 2020; Rook et al., 2021). A recent study has provided insights into the role of ASIC currents in the generation of action potential (AP) firing in neurons in relation to the speed of pH change. The authors demonstrate that moderate acidification (4–10 s) mediates AP increase, while slow acidification (>10 s) is not able to induce AP changes, suggesting a new important parameter that influences ASIC modulation of neuronal depolarization (Alijevic et al., 2020).

Most studies are focused on the most abundant form of an ASIC channel expressed in the CNS, which is represented by ASIC1a. Activation of ASIC1a plays a key role in pathological processes by promoting neuronal death *via* calcium (Ca^2+^)-mediated toxicity (Yermolaieva et al., 2004). The abundant expression of ASIC1a in discrete regions of the brain, such as the hippocampus, cortex, striatum, and amygdala (Alvarez de la Rosa et al., 2003; Wemmie et al., 2003; Coryell et al., 2009) and the characteristic to be Ca^2+^ permeable has suggested a role for this channel in calcium-related processes such as synaptic plasticity, learning and memory, as well as neuronal death (Wemmie et al., 2002; Gao et al., 2015; Wang et al., 2015). Accordingly, blocking ASIC1a affects multiple forms of synaptic plasticity in many brain regions including the amygdala and hippocampus (Wemmie et al., 2002, 2003; Du et al., 2014; Mango et al., 2017; Mango and Nisticò, 2020). Inhibition of ASIC1a produces a reduction in magnitude of both forms of synaptic plasticity long-term potentiation (LTP) and long-term depression (LTD) and ASIC1a KO mice show impairment of learning and memory evaluated with the Morris water maze and elevated-plus maze behavioral tests.

Moreover, the interplay between ASIC1a and NMDA, AMPA, and GABA receptors represents a potential target for acidotoxicity and exitotoxicity that occur in pathological conditions, such as ischemia (Gao et al., 2005, 2016; Chen et al., 2011; González-Inchauspe et al., 2017). Intriguingly, protons are able to modulate different synaptic receptors including AMPA and NMDA glutamate receptors (Tang et al., 1990; Traynelis and Cull-Candy, 1990; Lei et al., 2001), as well as GABAA receptors (Kaila, 1994; Dietrich and Morad, 2010), indicating that pH changes affect neuronal excitability, neurotransmission, and post-synaptic responses (Chen and Chesler, 1992; Makani and Chesler, 2007; Dietrich and Morad, 2010).

## Neuroinflammation Process and pH Shift in Neurodegenerative Disorders

Growing evidence supports the relationship between neuroinflammation and synaptic degeneration as a common feature in neurodegenerative diseases including AD, PD, and MS (Nisticò et al., 2017; Guzman-Martinez et al., 2019). Acidosis and accumulation of protons in the extracellular space represent hallmarks of the inflammatory process (see Diaz et al., 2018). The shift to the acidic pH was observed in normal aging and in patients affected by a variety of neurodegenerative disorders and was also confirmed in experimental models of neuroinflammation (Roberts and Sick, 1996; Roberts and Chih, 1997; Friese et al., 2007; Vergo et al., 2011; Mandal et al., 2012; Tyrtyshnaia et al., 2016).

To date, there is limited experimental evidence linking ASICs to neuroinflammation. For example, NSAIDs were reported to act against ASICs by both preventing inflammation-induced increase in expression and directly inhibiting the channels (Voilley et al., 2001). Our group has published that CHF5074 (also called CSP-1103), a flurbiprofen derivative, induces inhibition of ASIC1a-mediated currents elicited by application of pH 5.5 bath medium (IC50 ∼50 nM) (Mango et al., 2014). A previous work has also demonstrated an increased expression of ASIC3 on nerve afferents supplying joints in response to inflammatory stimulus and an anti-inflammatory action exerted by ASIC3 inhibitors in animal models of rheumatoid arthritis. Notably, ASIC^–/–^ animals manifest higher joint inflammation and destruction compared with ASIC^+/+^ controls (Sluka et al., 2013).

Therefore, ASICs represent the receptors on which the protons act as neurotransmitter and prolonged changes in pH shift likely contribute to the chronic inflammatory states underlying the neurodegenerative process.

### Alzheimer’s Disease

AD is a major cause of dementia in the world population. AD is characterized by chronic neurodegeneration with progressive memory impairment (Chapman et al., 1999), and currently, there is limited effective treatment or cure.

Several studies highlighted a shift to acidic pH in the AD brain caused by reduced cerebral perfusion (Siesjo, 1988). Changes in pH have also been observed in cerebrospinal fluid and post-mortem brain tissue of AD patients (Liu et al., 1999; Preece and Cairns, 2003).

ASIC1a plays a key role in synaptic plasticity processes including neurotransmission, dendritic structural remodeling, learning and memory, and fear response. This has been widely demonstrated in non-clinical studies using electrophysiological and behavioral approaches in mice (Wemmie et al., 2002, 2003; Chu and Xiong, 2012; Huang et al., 2015; Mango and Nisticò, 2019).

However, the role of ASIC1a in the synaptic alterations underlying AD remains still elusive. Indeed, our group has previously demonstrated an involvement of ASIC1a in hippocampal LTD ion in two experimental models of AD (Mango and Nisticò, 2018). Specifically, blocking ASIC1a with the selective blocker psalmotoxin-1 inhibited the enhancement of mGlu receptor-dependent LTD seen in the hippocampal slices, which were either treated with Aβ oligomers or obtained from Tg2576 mouse model of AD (Mango and Nisticò, 2018). The significance of these results remains to be established, and further studies need to be conducted in order to clarify the ASIC1a involvement in AD pathophysiology.

### Parkinson’s Disease

PD is a neurodegenerative disorder characterized by progressive and selective degeneration of dopaminergic neurons localized in the substantia nigra pars compacta (SNpc) area of the brain and cytoplasmic inclusions of alpha-synuclein aggregates, called Lewy bodies (Gelders et al., 2018). The neurodegeneration is responsible for motor and non-motor symptomatology including tremor, rigidity, bradykinesia, olfactory dysfunction, sleep disturbance, and cognitive impairment (Gelders et al., 2018).

Several studies described an elevated concentration of neuroinflammatory markers in PD patients suggesting the hypothesis that inflammation plays a role in neurodegeneration (Gelders et al., 2018). Also, PD is associated with lactic acidosis, and a study performed using an animal model of PD, the MPTP-treated mouse, described an involvement of ASIC1a in neurodegeneration of dopaminergic neurons. Indeed, blocking ASIC1a with amiloride or psalmotoxin-1 preserved dopaminergic neurons of SNpc from degeneration (Arias et al., 2008). Mutations in *Parkin* gene associated with autosomal recessive juvenile onset of PD (Kitada et al., 1998) or lack of the Parkin gene facilitate ASIC1a currents in hippocampal neurons evoked by application of acidic extracellular solution, and increase vulnerability of dopaminergic neurons, suggesting a significant role of ASIC1a in PD pathophysiology (Joch et al., 2007).

Nevertheless, not many studies have been performed to verify the role of ASIC1a as a therapeutic target for PD.

### Multiple Sclerosis

MS is a chronic autoimmune inflammatory disease characterized by demyelination and axonal degeneration processes (Goldenberg, 2012).

Many studies investigated the involvement of ASIC1a in MS using a well-accepted animal model of MS that is the experimental autoimmune encephalomyelitis (EAE) model (Friese et al., 2007; Bjelobaba et al., 2018). Previous studies found ASIC1a upregulation in axons and oligodendrocytes in the EAE model and in MS patients (Vergo et al., 2011). In MS patients, a relationship between elevated ASIC1a expression and axon injury markers was demonstrated, and this was also confirmed in EAE mice (Vergo et al., 2011; Arun et al., 2013). Also, administration of ASIC blocker amiloride attenuated demyelination and neuronal damage in EAE animal model as well as in a cohort of MS patients (Arun et al., 2013), indicating that ASIC1a inhibition is neuroprotective and represents a promising therapeutic strategy for MS.

### Huntington’s Disease

HD is a rare hereditary neurodegenerative disease characterized by movement and cognitive disorders and polyglutamine repeat as pathologic hallmarks (Agostinho et al., 2013). Not much evidence is present in literature linking ASIC1a to HD. The acidosis that occurs in the brain of HD patients and in mouse models of HD suggests an involvement of these channels in the pathophysiology of HD (Tsang et al., 2006; Josefsen et al., 2010). Notably, blocking ASIC1a with an amiloride derivative benzamil was able to alleviate pathology through reduction of Huntingtin–polyglutamine aggregation, the HD hallmark in the striatum of R6/2 mice model of HD (Wong et al., 2008).

## Acid-Sensing Ion Channel Modulators and Their Potential Therapeutic Use

Experimental evidence suggests that ASIC1a could represent a new therapeutic target for neurodegenerative diseases. Below, we present the main ligands targeting ASIC1a, and the binding sites of ASIC1a are illustrated in Figure 1.

### Amiloride

Amiloride a potassium-sparing diuretic agent and acts as a non-specific ASIC1a inhibitor in the range of 1–100 μM, which act by binding to the ion pore (Schild et al., 1997; Leng et al., 2016). Clinical studies have detected a neuroprotective and myeloprotective effect in patients with primary progressive MS (Arun et al., 2013). Nevertheless, amiloride was not effective in reducing the brain atrophy in patients with secondary progressive multiple sclerosis (De Angelis et al., 2020). In the light of these data, it could be important to test other amiloride analogs, such as benzamil with increased potency and selectivity for ASIC1a (Leng et al., 2016).

### Non-steroidal Anti-inflammatory Drugs

Non-steroidal anti-inflammatory drugs (NSAIDs) are a class of drugs widely used for the treatment of inflammation, pain, and many other disorders (Voilley, 2004). Previous studies have demonstrated that flurbiprofen, ibuprofen, and derivates mediate a direct inhibition of ASIC currents acting on the β-ball domain, with a significant reduction in evoked currents by the application of acidic medium (Voilley et al., 2001; Mango et al., 2014).

### Small Molecules

Different small molecules targeting ASICs were developed and represent new pharmacological tools used to study the ASICs function. Some of these molecules are clinically tested for therapeutic use, as PPC-5650 for the irritable bowel syndrome (Dubé et al., 2009; Olesen et al., 2015).

In this class can be cited NS-383 small potent inhibitor of ASIC1a (Munro et al., 2016) and two potent allosteric antagonists of ASIC1a JNJ-799760 and JNJ-67869386, which act by binding to the acidic pocket (Liu et al., 2021). Other new potent antagonists are represented by A-317567 and 5b (Dubé et al., 2005; Buta et al., 2015).

### Psalmotoxin-1

Psalmotoxin-1 (PcTx-1) was the first toxin identified as a selective ASIC1a inhibitor, which acts by binding to an acidic pocket (Escoubas et al., 2000). Although, the structure of PcTx-1 does not allow passage through the blood–brain barrier, previous studies demonstrated its neuroprotective effect in different experimental models of neurodegenerative diseases *via* intrathecal or intracerebroventricular administration (Diochot et al., 2012; Baron et al., 2013; Chassagnon et al., 2017).

### ASC06-IgG1

ASC06-IgG1 is a novel selective ASIC1a-blocking monoclonal antibody, which showed potent, sustained, and highly selective inhibition of ASIC1a (Qiang et al., 2018). This specific antibody shows a neuroprotective effect against ischemia (Qiang et al., 2018). Further studies are needed to investigate the neuroprotective effect of ASC06-IgG in experimental models of neurodegenerative diseases. Today, numerous monoclonal antibodies have been approved or are under development for neurological diseases (Sirbu et al., 2021), but many limitations, including the route of administration, as well as class-specific and target-associated risks, have limited their use (Loureiro et al., 2014, 2017; Teleanu et al., 2019).

## Conclusion

Neurodegenerative diseases are characterized by different and multifactorial processes sharing accumulation of misfolding proteins, damage of specific neuronal populations, and chronic inflammation state neuroinflammation. Another common feature is the shift to the acidic pH in the brain, which might contribute to apoptosis, protein misfolding, excitotoxicity, and neurodegeneration (Parton et al., 1991; Bender et al., 1997; Ding et al., 2000; Liu et al., 2012; Wang et al., 2012).

Emerging evidence supports the role of ASIC activation in several physiological processes such as synaptic plasticity and memory. On the other hand, ASICs are implicated in the pathophysiology of inflammatory and neurodegenerative diseases including PD, MS, AD, and HD.

The complex molecular pathways leading to neurodegeneration makes the search for novel effective agents difficult. In this frame, the development of potent and specific blockers for individual ASIC subunits, as well as the targeting of endogenous signaling molecules modulating the function of ASICs, might represent a new approach for the treatment of neurodegenerative conditions.

## Author Contributions

DM conceived the idea and prepared the manuscript. RN reviewed the drafts. Both authors contributed to the writing and approved final version of the manuscript.

## Conflict of Interest

The authors declare that the research was conducted in the absence of any commercial or financial relationships that could be construed as a potential conflict of interest.

## Publisher’s Note

All claims expressed in this article are solely those of the authors and do not necessarily represent those of their affiliated organizations, or those of the publisher, the editors and the reviewers. Any product that may be evaluated in this article, or claim that may be made by its manufacturer, is not guaranteed or endorsed by the publisher.
